# Identification of icaritin derivative IC2 as an SCD-1 inhibitor with anti-breast cancer properties through induction of cell apoptosis

**DOI:** 10.1186/s12935-022-02621-y

**Published:** 2022-05-31

**Authors:** Chen Yang, Yi-Yuan Jin, Jie Mei, Die Hu, Xiaoyu Jiao, Hui-Lian Che, Chun-Lei Tang, Yan Zhang, Guo-Sheng Wu

**Affiliations:** 1 Wuxi School of Medicine, Jiangnan University, Wuxi, 214000 Jiangsu China; 2 Department of Oncology, Wuxi Maternal and Child Health Hospital Affiliated to Nanjing Medical University, Wuxi, 214000 China; 3 Wuxi Clinical Medical College, Nanjing Medical University, Wuxi, 214000 China; 4 Jiangsu Key Laboratory of Advanced Catalytic Materials & Technology, School of Petrochemical Engineering, Changzhou, 213164 China; 5 School of Pharmaceutical Science, Jiangnan University, Wuxi, 214000 China; 6 Taizhou Center for Disease Control and Prevention, Taizhou, 318000 China

## Abstract

**Background:**

Breast cancer is the most common malignancy affecting women, yet effective targets and related candidate compounds for breast cancer treatment are still lacking. The lipogenic enzyme, stearoyl-CoA desaturase-1 (SCD1), has been considered a potential target for breast cancer treatment. Icaritin (ICT), a prenylflavonoid derivative from the Traditional Chinese Medicine *Epimedii Herba*, has been reported to exert anticancer effects in various types of cancer. The purpose of the present study was to explore the effect of the new ICT derivative, IC2, targeting SCD1 on breast cancer cells and to explore the specific mechanism.

**Methods:**

Immunohistochemistry and semiquantitative evaluation were performed to detect the expression level of SCD1 in normal and tumor samples. Computer-aided drug design (CADD) technology was used to target SCD1 by molecular docking simulation, and several new ICT derivatives were prepared by conventional chemical synthesis. Cell viability was evaluated by an MTT assay and dead cell staining. SCD1 expression in cancer cells was determined by Western blot and qRT-PCR analyses. The enzymatic activity of SCD1 was evaluated by detecting the conversion rate of [d31] palmitic acid (PA) using Gas chromatography-mass spectrometry (GC–MS). DAPI staining, flow cytometry and Western blot were used to detect cell apoptosis. Mitochondrial membrane potential and reactive oxygen species (ROS) assays were used to determine cell mitochondrial function. Lentiviral transduction was utilized to generate SCD1-overexpressing cell lines.

**Results:**

We found that SCD1 was overexpressed and correlated with poor prognosis in breast cancer patients. Among a series of ICT derivatives, in vitro data showed that IC2 potentially inhibited the viability of breast cancer cells, and the mechanistic study revealed that IC2 treatment resulted in ROS activation and cellular apoptosis. We demonstrated that IC2 inhibited SCD1 activity and expression in breast cancer cells in a dose-dependent manner. Moreover, SCD1 overexpression alleviated IC2-induced cytotoxicity and apoptosis in breast cancer cells.

**Conclusions:**

The new ICT derivative, IC2, was developed to induce breast cancer cell apoptosis by inhibiting SCD1, which provides a basis for the development of IC2 as a potential clinical compound for breast cancer treatment.

## Introduction

According to the latest global cancer data, breast cancer has surpassed lung cancer to become the leading cause of cancer incidence. Moreover, breast cancer ranks first in terms of mortality in women [1]. Although the combination of radiotherapy, surgery and chemotherapy/hormonotherapy has been broadly employed in breast cancer therapy, women still experience varying side effects that influence their health-related quality of life [2, 3]. Therefore, it is necessary to identify novel therapeutic targets and to develop related candidate compounds for improving the efficacy of breast cancer therapy.

Because tumors are characterized by uncontrolled cell growth and proliferation, they require additional nutritive material and energy requirements [4]. To meet these needs, the metabolism of tumor cells undergoes tremendous changes, such as de novo fatty acid synthesis. SCD1, an iron-containing endoplasmic reticulum-bound enzyme, is a key participant in de novo fatty acid synthesis. SCD1 catalyzes the conversion of endogenous and exogenous saturated fatty acids (SFAs) into monounsaturated fatty acids (MUFAs) and cooperates with other lipogenic enzymes, such as ACC and FASN, to participate in lipid synthesis [5]. As the main product of SCD1, oleic acid (OA) has been reported to protect cells from lipotoxicity [3, 6]. Compared to silencing other lipogenic genes, silencing SCD1 in breast cancer cells exerts the strongest inhibitory effect on cell proliferation and a weaker inhibitory effect on homologous nonmalignant epithelial cells [4, 5, 7, 8]. Therefore, SCD1 represents a promising target for breast cancer treatment. A variety of small molecule inhibitors targeting SCD1 have been developed with significant antitumor effects, such as T-3764518, CAY-10566 and MF438 [5]. However, obvious toxic effects have been observed after SCD1 inhibitor treatment in vivo, thereby preventing SCD1 inhibitors from entering clinical trials as anticancer drugs [5]. Therefore, the active ingredients from traditional Chinese medicine, characterized by less toxicity, may be a good option to be developed as SCD1 inhibitor candidates and used as an adjuvant for breast cancer treatment.

ICT is a major ingredient from the Chinese herbal medicine, *Epimedii Herba (Yinyanghuo)*, which has been used in Chinese medicine for centuries [9]. Previous studies have demonstrated that ICT has a wide range of biological and pharmacological functions, including estrogen-like activity, neuroprotection, osteogenesis, immunomodulation and cardiac differentiation stimulation [10–14]. Recently, emerging evidence suggests that ICT can be used against different types of cancer cells by inducing apoptosis, cell cycle arrest and immune responses [11]. ICT promotes cell apoptosis by downregulating AFP gene expression in hepatocellular carcinoma [15], and it suppresses the proliferation and invasion of prostate cancer cells by downregulating UBE2C expression and upregulating miR‐381‐3p levels [16]. In breast cancer cells, studies have shown that ICT induces cell cycle arrest and apoptosis through the continuous activation of ERK, decreasing the expression of ER-α36 and EGFR protein [17, 18]. Furthermore, ICT has also been found to regulate lipid metabolism by inhibiting adipocyte differentiation and downregulating adipogenic transcription factors [19]. At present, a large phase III clinical trial of ICT for the treatment of hepatocellular cancer has been completed in China, suggesting a good safety and therapeutic effect of ICT on hepatocellular carcinoma, and another clinical trial of ICT in breast cancer is also underway [20]. Therefore, we hypothesized that the development of ICT and its derivatives targeting lipid metabolism is beneficial for breast cancer treatment.

In the present study, we used CADD simulation to modify the ICT group, and we screened out the potential SCD1 inhibitor, IC2, and conducted a preliminary exploration of its potential mechanism. We demonstrated that IC2 promotes the apoptosis of breast cancer cells by inhibiting SCD1, indicating the potential of this compound as a drug candidate for breast cancer treatment.

## Materials and methods

### Cell culture and materials

MCF-7, SK-BR-3, and 293T cells were obtained from the Cell Bank of Shanghai Institutes for Biological Sciences, Chinese Academy of Sciences. MCF-7, SK-BR-3, and 293T cells were cultured in Dulbecco’s modified Eagle’s medium (DMEM) with 10% FBS, 100 U/mL penicillin and 100 mg/mL streptomycin. Cells were maintained at 37 °C in a humidified environment in an incubator with 5% CO2.

ICT was purchased from Nanjing spring&autumn biotech co.ltd (Cat: E-0846, Nanjing, China). DMEM containing high glucose and fetal bovine serum (FBS) was purchased from HyClone (Cat: SH30022, Logan, Utah, USA). Reactive Oxygen Species Assay Kit (Cat: S0033), mCherry-GFP-LC3B (Cat: 3011) and JC-1(Cat: C2055) were obtained from by Beyotime (Shanghai, China). The MTT assay kits (Cat: MB4698) and DAPI solution (Cat: MA0128) were purchased from Meilunblo (Dalian, China). The FITC Annexin V Apoptosis Detection Kit was obtained from BD Biosciences (Cat: 556547, Becton Dickinson and Company, San Jose, CA, USA), and the LIVE/DEADTM Fixable Green Dead Cell Stain Kit was purchased from Thermo Scientific (Cat: L34970, Carlsbad, CA, USA). All primary antibodies, including anti-PARP (Cat: 13038P), anti-Bax (Cat: 5023S), anti-Bcl-2 (Cat: 4223T), anti-β actin (Cat: 4970T) and anti-SCD1 (Cat: 12438S), as well as the secondary antibody (Cat: 7074T) were purchased from Cell Signaling Technology (Beverly, MA, USA). The primary antibody, anti-Flag (Cat:20543–1-AP), was purchased from Proteintech (Wuhan, China). Oleic acid (OA, Cat: A63262) and palmitic acid (PA, Cat: 81914) were purchased from Sinopharm (Shanghai, China). [d31]PA was purchased from Cayman (Cat: 16497, Ann Arbor, MI, USA). BSA was obtained from BioFroxx (Cat: 143064, Einhausen, Germany). All reagents used in the experiments were of analytical grade or higher.

### Clinical samples

The breast cancer tissue microarray (TMA) section (HBre-Duc159Sur-01) was purchased from Outdo Biotech (Shanghai, China). The section contained 40 normal or benign breast samples and 119 breast cancer samples. The detailed clinicopathological characteristic records and follow-up data for each sample were also provided by Outdo Biotech. Ethical approval (YB-M-05-02) for the study of the TMA was granted by the Clinical Research Ethics Committee, Outdo Biotech (Shanghai, China).

### Immunohistochemistry and semiquantitative evaluation

To evaluate the expression level of SCD1 in normal and tumor samples, immunohistochemistry (IHC) staining was performed using an anti-SCD1 antibody (1:1000 dilution, Cat. 23393-1-AP, ProteinTech, Wuhan, China) and the TMA according to standard processes. The TMA was scanned using Aperio Digital Pathology Slide Scanners. For semiquantitative evaluation of SCD1 expression, all samples were independently assessed by two independent pathologists based on the immunoreactivity score (IRS) strategy as previously described [21]. Briefly, the percentage of positively stained cells was scored as 0–4 as follows: 0 (< 5%), 1 (6–25%), 2 (26–50%), 3 (51–75%) and 4 (> 75%). The staining intensity was scored as 0–3 as follows: 0 (negative), 1 (weak), 2 (moderate) and 3 (strong). The IRS equals the score of the percentage of positive cells multiplied by the score for staining intensity.

### Kaplan–Meier plotter analysis

To validate the prognostic value of SCD1 in breast cancer, SCD1 (Affy ID: 200832_s_at, JetSet best probe) mRNA expression data and survival data were downloaded from Kaplan–Meier plotter (http://kmplot.com). Kaplan–Meier survival plots were then generated, and survival curves were compared by the log-rank test.

### Molecular docking simulation

The three-dimensional (3D) structure of human stearoyl–coenzyme A desaturase 1 (hSCD1) (PDB ID: 4ZYO) was downloaded from the protein data bank (https://www1.rcsb.org/). The 3D structure of hSCD1 was subjected to clean water and ligand molecules using Discovery Studio 2018 software (https://www.3dsbiovia.com/). The 3D structures of ICT, IC1, IC2 and IC3 were handled and minimized by the CHARMm force field in Discovery Studio 2018. Molecular docking simulations of hSCD1 and ligand ICT, IC1, IC-2 or IC-3 were performed with a flexible ligand by a genetic algorithm using AutoDock vina (https://autodock.scripps.edu/) [19]. Molecule-docked complexes of hSCD1–ICT, hSCD1–IC1, hSCD1–IC2 and hSCD1–IC3 were added to a CHARMm force field. Two stages of energy minimization of the fixed and unfixed with protein and ligand were conducted to relax internal constraints by a 1000-step steepest descent algorithm and a 2000-step conjugate gradient algorithm, respectively. The interaction energy, binding free energies (ΔGbind) and nonbinding interactions were analyzed according to the minimized molecule-docked complexes by Discovery Studio 2018 software.

### Preparation of ICT derivatives

ICT and 1-bromo-3-methyl-2-butene were dissolved in 150 mL of acetone. Then, 300 mg of anhydrous potassium carbonate was added to the mixture. The reaction mixture was heated to 56 ℃ and stirred for 4 h. The reaction mixture was then concentrated under reduced pressure, adjusted to pH 4–5 with 1 N HCl and then extracted three times with 100 mL of dichloromethane. The organic extracts were dried over anhydrous sodium sulfate, filtered and concentrated under reduced pressure. The resulting residue was purified by silica gel column chromatography (petroleum ether: ethyl acetate = 10:1) to obtain IC1, IC2 and IC3.

### MTT assay

MCF-7 cells were seeded in a 96-well microplate overnight and incubated with compounds at different concentrations for 24 h. The 3-(4,5-dimethylthiazol)-2,5-diphenyltetrazolium bromide (MTT) assay was used to calculate cell viability as described previously [22]. The results of the treated groups were normalized to the control group and then expressed as inhibition rates.

### Live and dead cell staining

The experiment was conducted according to the protocol of the LIVE/DEAD™ Fixable Dead Cell Stain Kit. For this kit, the fluorescent reactive dye permeates the compromised membranes of necrotic cells and reacts with cellular amines both in the interior and on the cell surface, resulting in intense fluorescent staining and discrimination between live and dead cells. After being exposed to IC2 at 0, 5, 10 and 20 μM, adherent and suspended cells were all collected and washed with PBS. One milliliter of cells (1 × 10^6^ in PBS) was stained with 1 mL of the fluorescent reactive dye and incubated at room temperature for 30 min (protected from light). One milliliter of PBS with 1% bovine serum albumin was used to wash the cells twice and resuspend the cells. The cell suspension was analyzed by flow cytometry (BD Biosciences, San Jose, USA) using 488 nm excitation.

### Western blot

Cell lysis buffer for Western blot and IP (Beyotime, Shanghai, China) containing protease inhibitors was used to extract proteins from cells, and the total concentration of proteins was determined using a BCA™ Protein Assay Kit (BIO-RAD, CA, USA). Protein samples were collected for electrophoresis with SDS–PAGE and then transferred to PVDF membranes (Bio-Rad, CA, USA). After blocking with 5% nonfat milk, the membranes were treated with primary antibodies (1:1000 dilution) and then secondary antibodies (1:1000–1:3000 dilution). Finally, membrane visualization was performed on an enhanced chemiluminescence (ECL) system (Thermo Scientific, Mannheim, Germany), and protein bands were detected using ChemiDOC MP (Bio-Rad, CA, USA). The density of the immunoreactive bands was quantified using ImageJ software (National Institute of Health, USA).

### Quantitative real-time PCR

After drug treatment, total RNA was isolated from MCF-7 cells using an RNAiso plus kit (Takara Bio Inc, Shiga, Japan) followed by reverse transcription to generate cDNA. Expression of mRNA was quantified using the LightCycler® 480 detection system (Roche Applied Science, Mannheim, Germany) with SYBR Green I Master according to the manufacturer’s instructions. The expression of target genes was analyzed by the 2-∆∆CT method with β-actin as an internal control. The following PCR primers were used: β-actin, 5′-TGACGTGGACATCCGCAAAG and 5′-CTGGAAGGTGGACAGCGAGG; SCD1, 5′-CCTCTACTTGGAGACGACATTCG and 5′-GCAGCCGAGCTTTGTAAGAGC.

### SCD1 activity assay

Cellular lipids were extracted using a modified version of the Bligh and Dyer method [23]. Aftercells were incubated with [d31]PA for 24 h, they were washed three times with PBS, and the cells were collected. Then, 4 mL of methanol, 2 mL of chloroform and 6 M hydrochloric acid were added to the cells, which were then shaken for 1 h. Then, 2 mL of chloroform and 2 mL of water were added followed by centrifugation at 2500 rpm for 10 min, and the lower layer was moved to a new fat-lifting bottle. The organic phase was dried in a nitrogen stream, and 1 mL of 0.5 M sodium hydroxide and methanol solution was added to the fat-lifting flask, which was then heated at 100 ℃ for 10 min to saponify the lipids. Then, 1 mL of 14% BF3 in methanol was added followed by heating at 100 ℃ for 10 min to prepare Fatty acid methyl esters (FAMEs). The sample was evaporated under nitrogen and resuspended in n-hexane. The FAMEs were separated and quantitatively analyzed by GC–MS, and the characteristics and content of the peaks were determined by detecting the characteristic ions in [d31]PA. The cell fatty acid profile was measured, and the ratio of [d31]C16:1/(C16:1 + C16:0), the conversion rate of [d31]PA, was used as an index to evaluate SCD1 activity.

### DAPI staining

Cells were seeded in 6-well plates overnight. After incubation with IC2 at different concentrations for 24 h, cells were washed three times in PBS, fixed with 4% formaldehyde for 10 min at room temperature and washed three times with PBS. Cells were then treated with 4,6-diamidino-2-phenylindole (DAPI; 2 µl/mL) for 20 min at 37 °C in the dark. After discarding the DAPI dye, cells were washed three times with PBS to remove the redundant fluorescent dye and then observed under a fluorescence microscope (Ti-U; Nikon, Tokyo, Japan), and images were acquired and analyzed.

### Annexin V-FITC/PI staining

To analyze apoptosis after treatment with IC2, an Annexin V-FITC/PI apoptosis detection kit (BD Biosciences, San Jose, CA, USA) was utilized. After treatment with IC2, cells were treated with Annexin V-FITC and PI in binding buffer for 30 min in the dark. Cells were analyzed by flow cytometry within 1 h. The early and late apoptotic cells were considered the apoptotic outcomes and were normalized to the control group.

### Mitochondrial membrane potential measurement

Cells were cultured in a 6-well plate overnight and then exposed to IC2 for 24 h. For microscopic observation, cells were incubated with a 5 mg/L JC-1 working solution for 25 min at 37 °C. After washing three times with PBS, cells were observed under an inverted fluorescence microscope (Nikon, Tokyo, Japan). Mitochondrial membrane potential (MMP) was also quantitatively measured using flow cytometry. After incubation with IC2, cells were collected and stained with a JC-1 working solution. The mixture was centrifuged, washed, resuspended and immediately analyzed with flow cytometry. JC-1 aggregates produce red fluorescence in the mitochondrial matrix when MMP is high. In contrast, JC-1 fails to aggregate and exists as a monomer with green fluorescence. Therefore, the ratio of the intensities of green and red fluorescence was used to compare the potential of the mitochondrial membrane.

### Reactive oxygen species measurement

The DCFH-DA fluorescent probe (Beyotime, Shanghai, China) was diluted in serum-free medium to 10 mM for cell staining following cell treatment with IC2 at different concentrations. After 30 min of staining, the cells were collected and resuspended in fresh medium for flow cytometry analysis.

### Lentiviral overexpression model

We used lentivirus to establish SCD1 overexpression models. The SCD1 overexpression lentiviral vector was purchased from Hanbio Biotech (Shanghai, China), and the lentiviral vector was transduced into MCF-7 cells according to the manufacturer's instructions. In short, cells were cultured in a 12-well plate and incubated overnight in a 37 °C and 5% CO2 incubator. Before infection, the virus liquid was thawed on ice in advance. After 24 h of infection, the virus-containing culture medium was replaced with fresh culture medium. The SCD1-overexpression vector carries the GFP reporter gene, flag-tagged SCD1 gene and the puromycin resistance gene. The negative control vector carries only GFP reporter gene, and the puromycin resistance gene. After 48 h of infection, the GFP expression efficiency of the cells was observed under a fluorescence microscope to evaluate the virus infection efficiency. Subsequently, the MCF-7 stable transgenic cell line was screened by puromycin (1 μg/mL), and finally determined by Western blot using Flag and SCD1 antibody.

### Statistical analysis

The prognostic value of SCD1 in breast cancer was evaluated by the log-rank test. All in vitro experiments were repeated at least three times, and the results are presented as the mean ± SD. Multiple groups were compared using one-way ANOVA followed by Bonferroni's multiple comparisons for every two groups. Data were analyzed with GraphPad Prism 7.0 (GraphPad Software, San Diego, CA, USA). *p* < 0.05 was considered statistically significant.

## Results

### SCD1 is overexpressed and correlates with poor prognosis in breast cancer

**Fig. 1:**
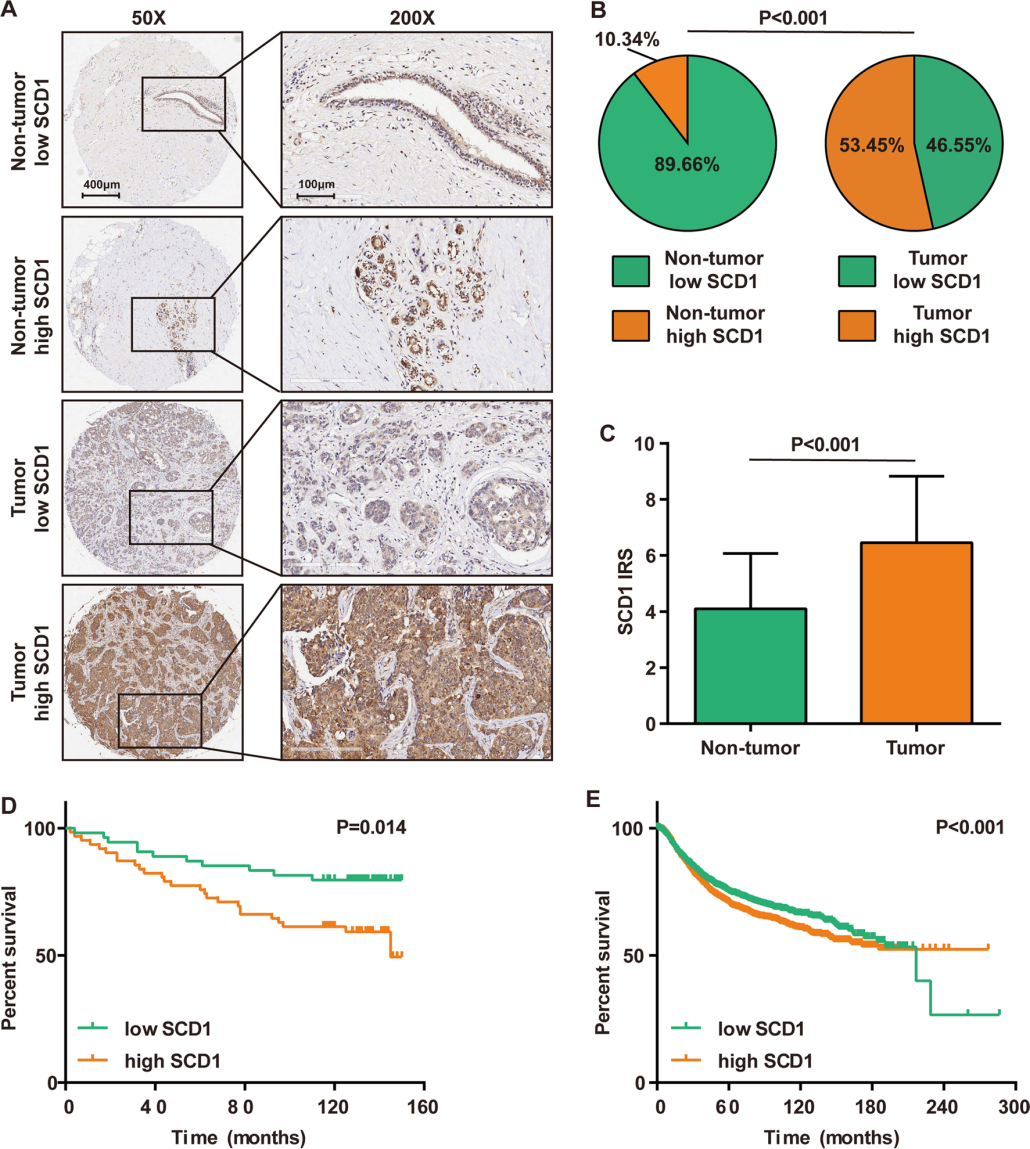
SCD1 expression in breast cancer and its prognostic value. **A** Representative microphotographs revealing SCD1 expression status using anti-SCD1 IHC staining. Brown, SCD1; Blue, hematoxylin. **B** The proportion of SCD1 expression status in tumor and nontumor samples. Low expression, IRS ≤ 6; High expression, IRS > 6. **C** Comparison of SCD1 expression in tumor and nontumor samples. **D** Survival analysis showing overall survival curves of patients with low SCD1 expression vs. high expression in breast cancer. Low expression, IRS ≤ 6; High expression, IRS > 6. **E** Online Kaplan–Meier Plotter analysis showing relapse-free survival curves of patients with low SCD1 expression vs. high SCD1 expression in breast cancer

Previous studies have revealed that SCD1 promotes the malignant biological properties of breast cancer cells via multiple signals [24, 25]. However, information about the expression of SCD1 in breast cancer and its consequences in patients is lacking. Therefore, SCD1 expression in breast cancer TMA samples was first analyzed by IHC staining. Most cells of the tumor samples were stained with anti-SCD1 antibodies, suggesting high or moderate expression levels of SCD1 (Fig. 1A, B). As a control, the majority of the cells in nontumor samples were not stained (Fig. 1A, B). Moreover, the results based on IRS scoring also indicated that the expression of SCD1 was remarkably enhanced in tumor tissues (Fig. 1C). Patients with high expression of SCD1 exhibited poorer prognosis than those with low SCD1 expression (Fig. 1D), and large-scale survival data from the Kaplan–Meier plotter also supported that high expression of SCD1 was correlated with poor prognosis in breast cancer (Fig. 1E). Taken together, the clinical data revealed that SCD1 is overexpressed in breast cancer and may be a novel therapeutic target for breast cancer.

### ICT derivatives mediate the inhibition of SCD1 activity by CADD

**Figure. 2.**
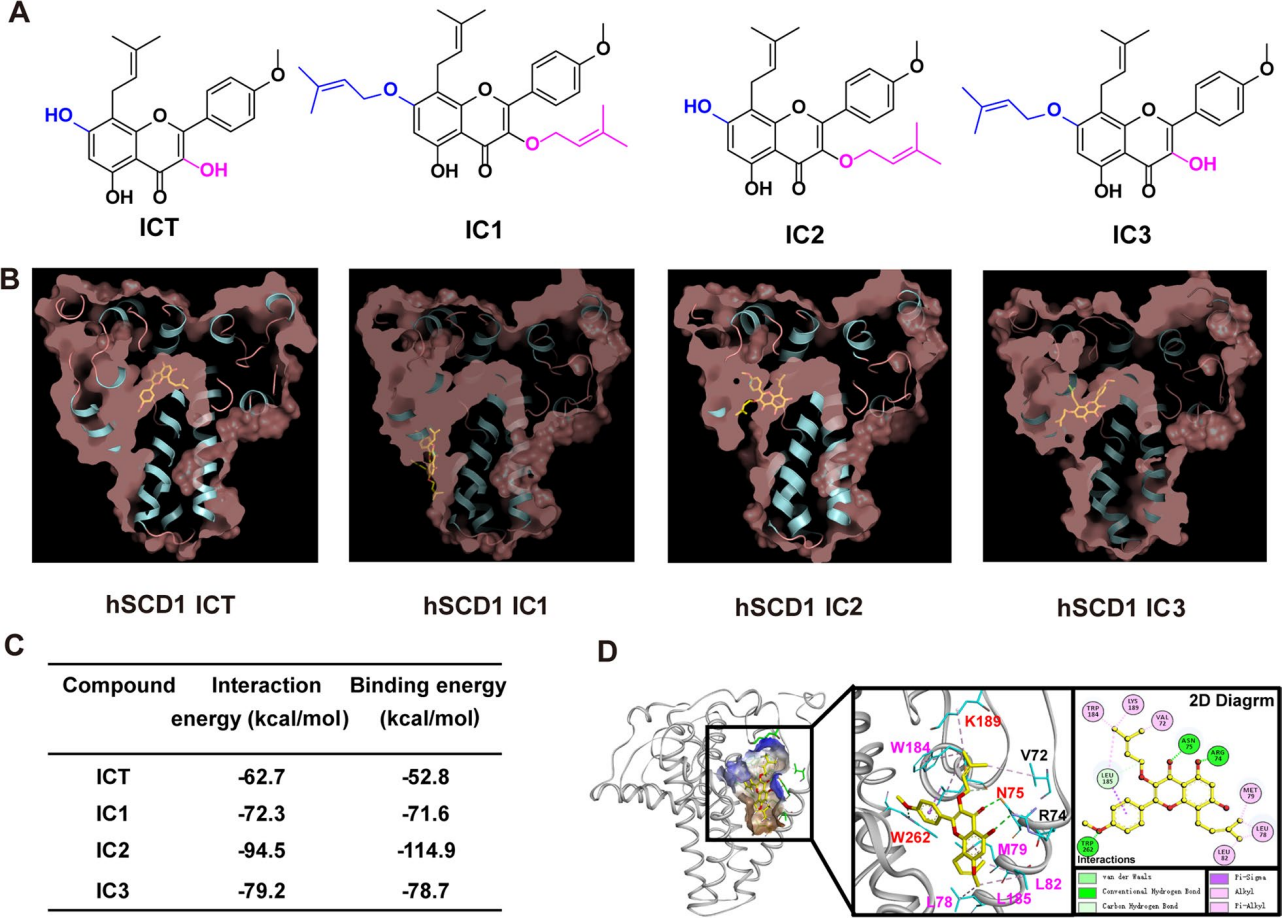
ICT derivatives mediate the inhibition of SCD1 activity and prediction of the binding mode by CADD. **A** The 2D structures of ICT, IC1, IC2 and IC3. **B** Cross-sections of the hSCD1 surface area (light red) docked with ICT, IC1, IC2 and IC3 (yellow sticks). **C** The interaction and binding energies of hSCD1 with ICT, IC1, IC2 and IC3. **D** The nonbinding interactions of hSCD1 with IC2. IC2 is depicted with yellow sticks, and interacting residues are depicted with cyan sticks. The hSCD1-IC-2 interactions are shown on a 2D diagram

Considering that *Epimedii Herba* extract and icariin have good performance in lipid accumulation inhibition [19], we hypothesized that ICT derivatives also affect cancer cell lipid metabolism. As shown in Fig. 2B, ICT, IC1, IC2 and IC3 docked into the long internal hydrophobic tunnel of hSCD1, while IC1 was outside of the tunnel as larger branched groups. Among the four molecule-docked complexes, IC2 had higher binding free energies (− 114.9 kcal/mol) than the other ICs (Fig. 2C). In addition, IC2 formed nonbinding interactions with 10 residues of hSCD1 with the following interactions: strong hydrogen bond interactions with R74, N75 and W262; alkyl interactions with V72, W184, K189, L78, M79 and L82; and van der Waals and Pi-sigma, Pi-alkyl interactions with L185. We also noted that N75, K189 and W262 (red letters) were the stearoyl-CoA–binding sites of hSCD1 [26] (Fig. 2D). These results indicated that IC2 binds to the stearoyl-CoA–binding pocket of hSCD1, thus inhibiting hSCD1 activity.

### Cytotoxicity of ICT derivatives on breast cancer cells

**Fig. 3:**
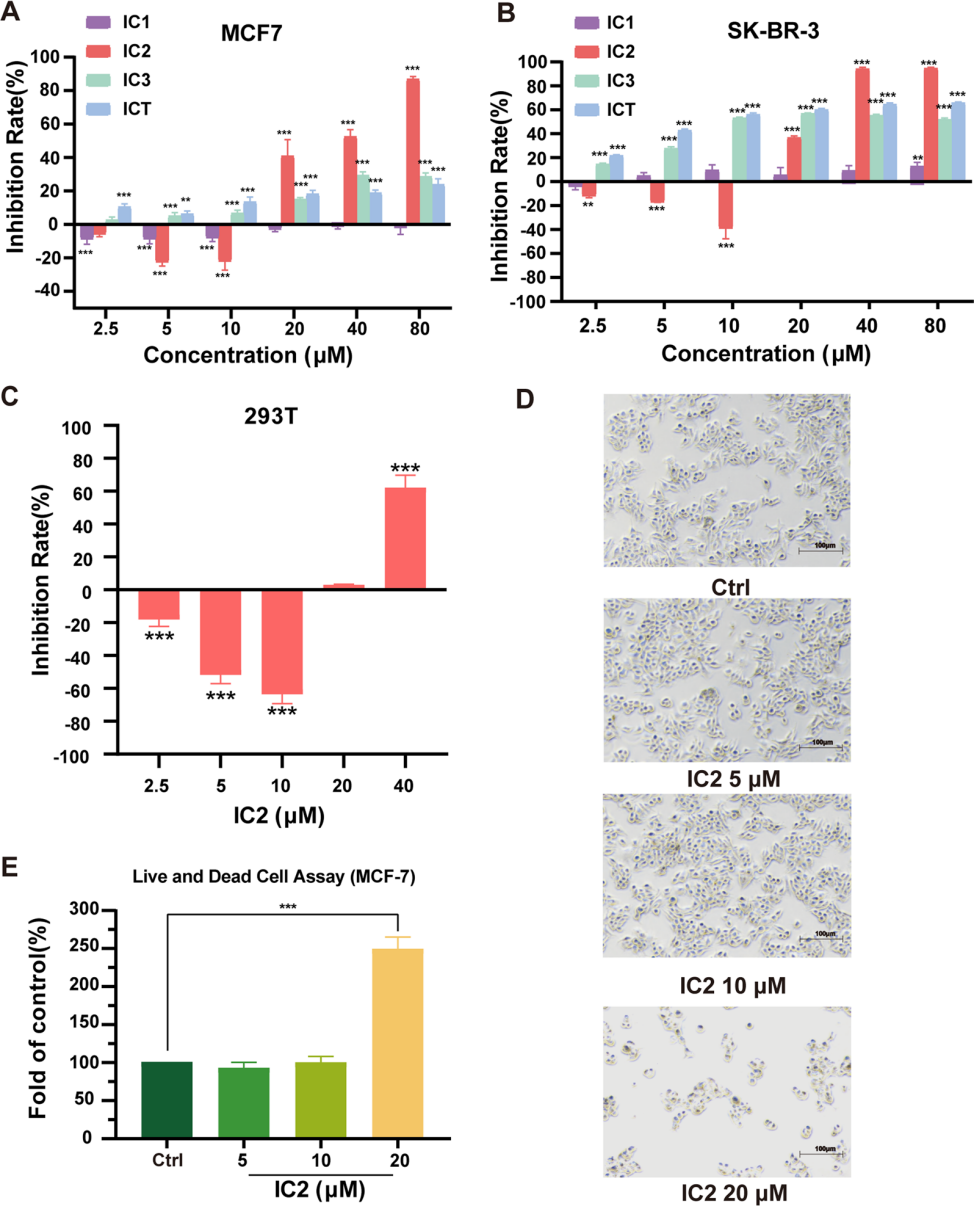
The antiproliferation effect of ICT derivatives in breast cancer cell lines. IC2 inhibited the growth of MCF-7 (**A**) and SK-BR-3 (**B**) cells to a greater extent than ICT and the other derivatives. **C** IC2 treatment (from 5 μM to 20 μM) for 24 h showed no cytotoxicity to normal human embryonic kidney cells (293T). **D** Observation under the microscope at ×50  magnification. Data are presented as the mean ± SEM of three independent experiments (n = 3). **E** LIVE/DEAD assay showed that the number of dead cells increased significantly after treatment with 20 μM IC2. *P < 0.05, **P < 0.01 and ***P < 0.001 vs. control

The effects of ICT derivatives on breast cancer cell viability for 24 h were investigated by MTT assays. IC2, IC3 and ICT inhibited cell growth in a dose-dependent manner. Among the three compounds, IC2 showed the most significant inhibitory effect at high concentrations (20 μM, 40 μM and 80 μM) in MCF-7 and SK-BR-3 cells (Fig. 3A, B). Consistent with the results of the MTT assay, observation under the microscope revealed that IC2 significantly inhibited cell growth at a concentration of 20 μM (Fig. 3D). LIVE/DEAD cell staining results also confirmed the anticancer effect of IC2 in MCF-7 cells (Fig. 3E). Compared to the control and low-dose groups, the number of dead cells in the treatment groups (20 μM) significantly increased. Besides, normal human embryonic kidney cells 293 T was used to detect the toxic effect of IC2. As shown in Fig. 3C, cell viability was not significantly affected in IC2-treated groups (2.5 μM to 20 μM) after incubation for 24 h. Thus, the concentrations used above and an incubation time of 24 h were applied in the following experiments.

### IC2 inhibits SCD1 expression and activity in breast cancer cells

**Fig. 4:**
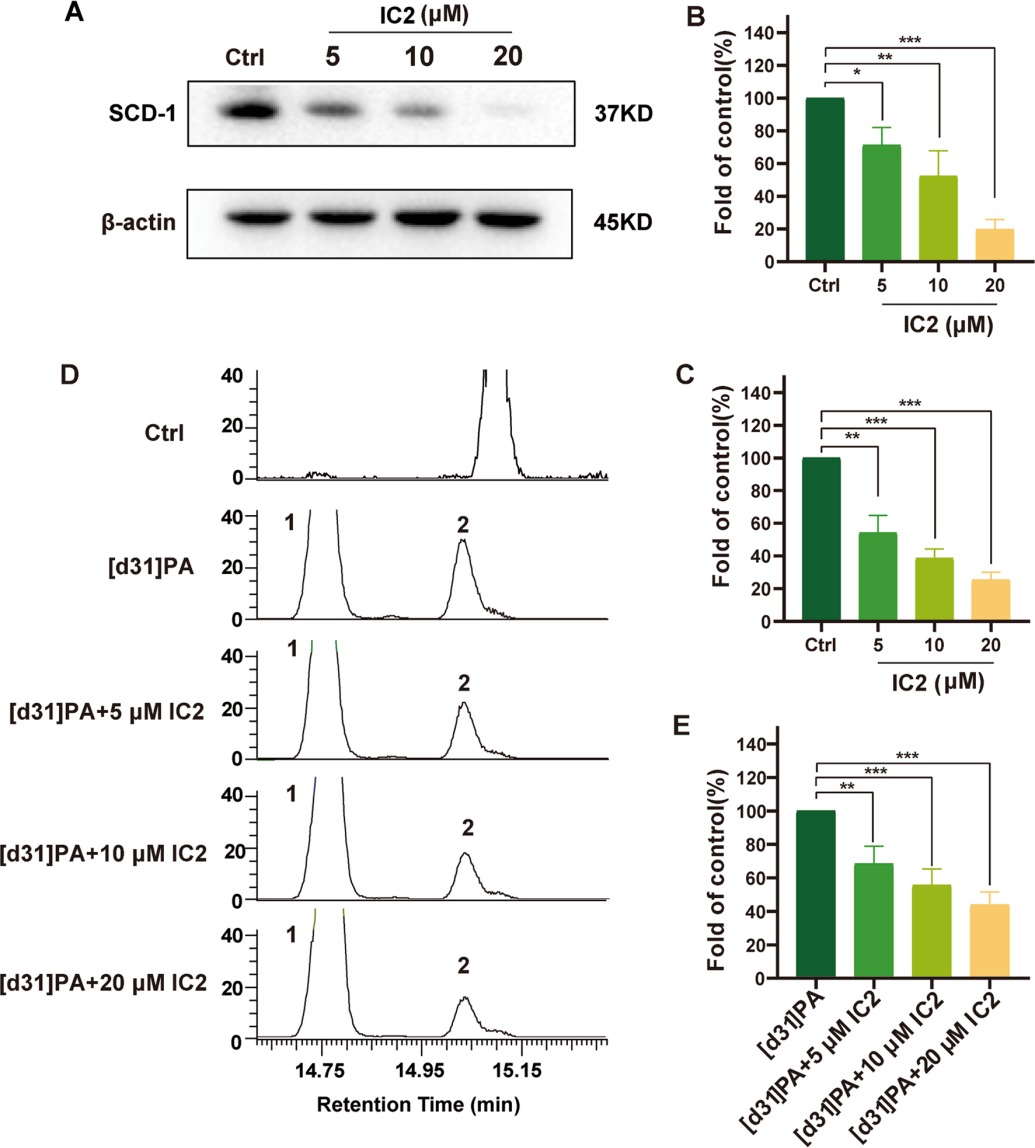
IC2 inhibits the expression and activity of SCD1 dose-dependently in MCF-7 cells. MCF-7 cells were treated with three concentrations of IC2 for 24 h, the protein (**A** and **B**), mRNA (**C**) and activity (**D** and **E**) levels of SCD1 were detected. (B) Quantification of the protein expression levels of SCD1. **E** Quantification of SCD1 activity levels. Data are presented as the mean ± SEM of three independent experiments (n = 3). *P < 0.05, **P < 0.01 and ***P < 0.001 vs. control

Because IC2 was a more effective inhibitor of cancer cell proliferation than the other ICT derivatives, IC2 was selected to further investigate the anticancer effects of ICT derivatives. As revealed by Western blot and qRT-PCR analyses, SCD1 expression was inhibited by IC2 at the mRNA and protein levels in a dose-dependent manner in MCF-7 cells (Fig. 4A–C). Because SCD1 mainly catalyzes the conversion of SFA into MUFA, we added isotope-labeled saturated fatty acid PA ([d31]PA) to detect the rate of the conversion of [d31]-labeled unsaturated fatty acid by GC–MS to reflect the change in SCD1 enzyme activity. After adding [d31]PA, IC2 treatment significantly reduced the conversion rate of [d31]-labeled unsaturated fatty acids in a dose-dependent manner, indicating that IC2 reduces SCD1 enzyme activity in a dose-dependent manner (Fig. 4D, E). The above results indicated that IC2 not only reduces SCD1 protein and gene expression but also inhibits SCD1 enzyme activity, verifying that IC2 acts as an SCD1 inhibitor.

### IC2 induces apoptosis of MCF-7 cells

**Fig. 5:**
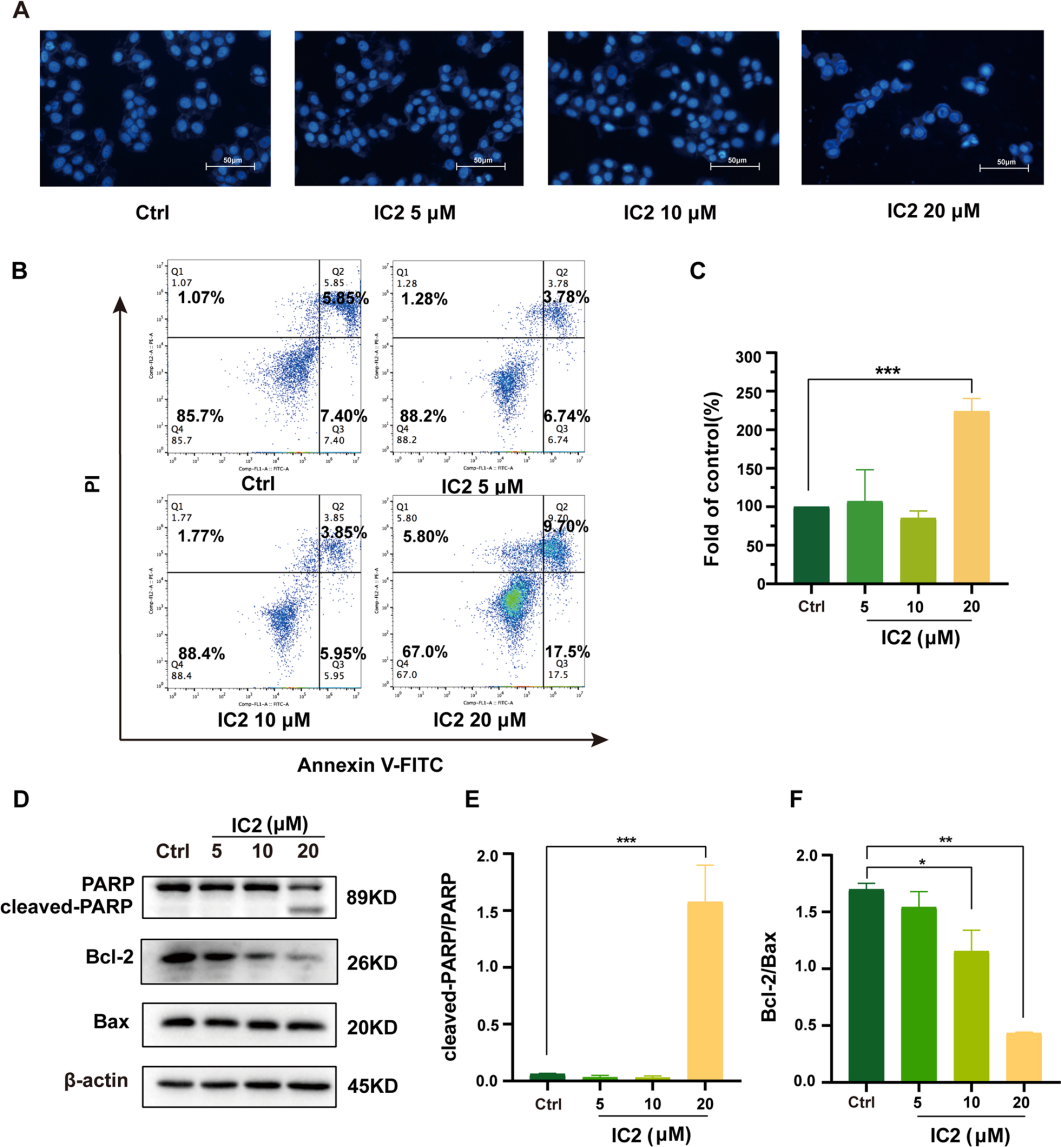
IC2 induces apoptosis of MCF-7 cells. MCF-7 cells were treated with three concentrations of IC2 for 24 h. **A** Representative images of DAPI staining at ×200 magnification in MCF-7 cells. **B** MCF-7 cells were double-stained with Annexin V and PI followed by flow cytometry analysis. The gate setting distinguished among living (bottom left), necrotic (top left), early apoptotic (bottom right) and late apoptotic (top right) cells. **C** Percentage of apoptotic cells compared to the control from **B**. **D** The expression of PARP, Bcl-2 and Bax was determined by Western blot. **E**, **F** Quantification of the protein expression levels of cleaved PARP/PARP and Bcl-2/Bax. Data are presented as the mean ± SEM of three independent experiments (n = 3). *P < 0.05, **P < 0.01 and ***P < 0.001 vs. control

Because high concentrations of IC2 exerted significant cytotoxic effects (Fig. 3C), we investigated the mechanisms of cell death induced by IC2. First, DAPI staining found that high concentrations of IC2 significantly reduced the number of live MCF-7 (Fig. 5A). The Annexin V-FITC/PI double staining method was then used to detect the content of apoptotic cells after IC2 treatment for 24 h. IC2 did not play a pro-apoptotic effect at concentrations of 5 μM and 10 μM, but the content of apoptotic cells increased significantly when the concentration of IC2 reached 20 μM or more, which was consistent with the previous results (Fig. 5A-C). As revealed by Western blot analysis, 20 μM IC2 promoted the expression of cleaved PARP in cell but decreased the expression of PARP (Fig. 5D, E). At the same time, IC2 inhibited the expression of Bcl-2 in a dose-dependent manner with minor effects on the expression of Bax (Fig. 5D, F).

### IC2 induces apoptosis of MCF-7 cells through the mitochondrial pathway

**Fig. 6:**
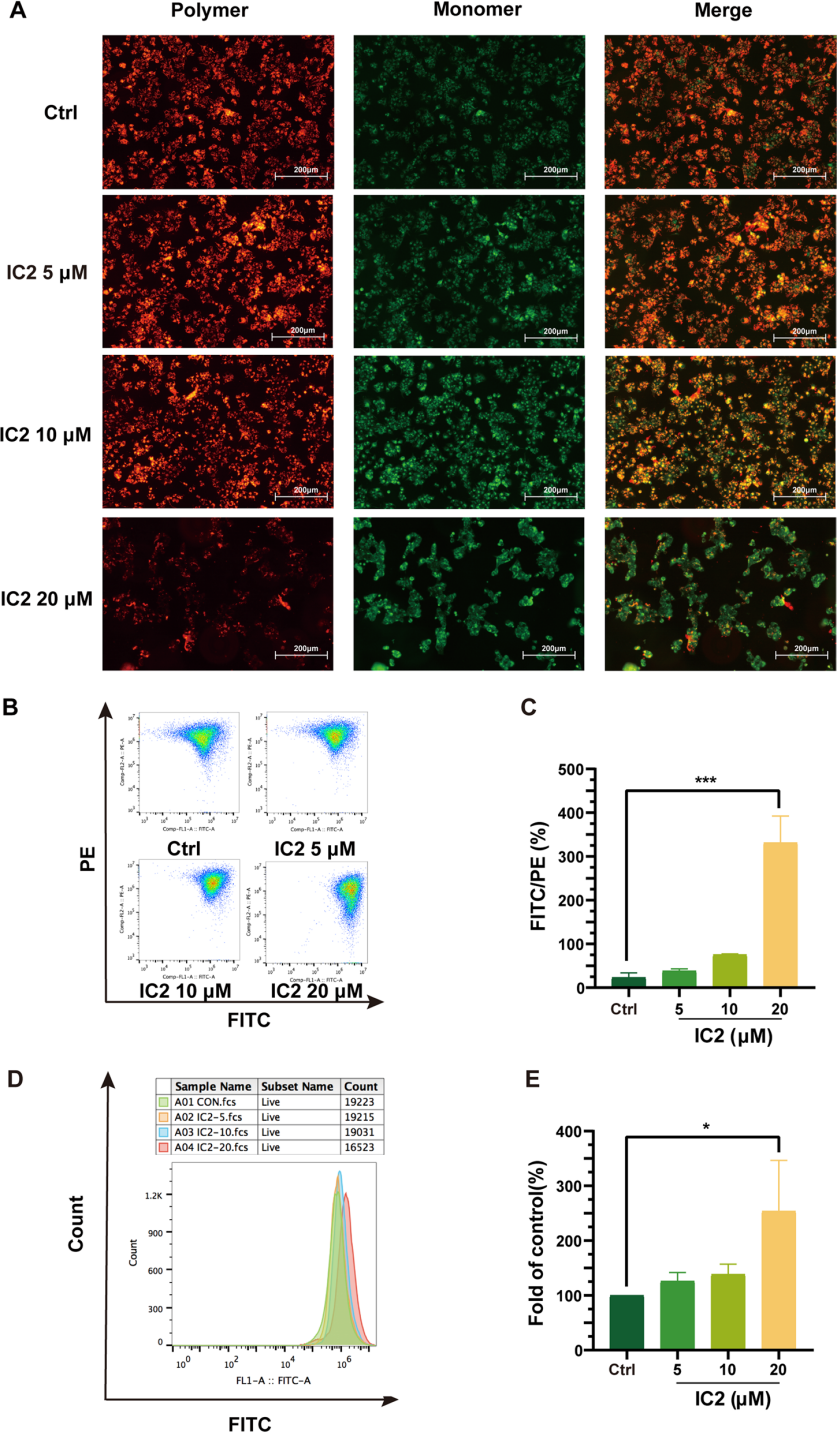
IC2 induces apoptosis of MCF-7 cells through the mitochondrial pathway. MCF-7 cells were treated with three concentrations of IC2 for 24 h. **A** Representative images of JC-1 staining at ×100 magnification in MCF-7 cells. **B** Flow cytometry was used to detect the deviation of red and green fluorescence in JC-1-stained cells. **C** Quantification of the ratio of red and green fluorescence in **B**. **D** Flow cytometry was used to detect intracellular ROS. **E** Quantification of the level of ROS production compared to the control in **D**. Data are presented as the mean ± SEM of three independent experiments (n = 3). *P < 0.05, **P < 0.01 and ***P < 0.001 vs. control

Cell apoptosis is achieved through the mitochondrial pathway, and the above results demonstrated that high concentrations of IC2 induce cell apoptosis. To further explore the IC2-induced apoptosis, the changes in mitochondrial membrane potential were detected by JC-I staining. After IC2 treatment, the red fluorescence intensity significantly decreased as shown by microscopy (Fig. 6A). At the same time, flow cytometry was applied to quantitatively analyze the fluorescence intensity. The cell population in the PE channel (red fluorescence) significantly shifted downward as the IC2 concentration increased, and the cell population in the FITC/PE channel (green fluorescence/red fluorescence) sharply increased, which indicated that the mitochondrial membrane potential significantly decreased after IC2 treatment (Fig. 6B, C).

Mitochondrial damage is often accompanied by the production of ROS. Therefore, we evaluated the level of ROS in cells treated with IC2. Flow cytometry analysis indicated that 5 μM and 10 μM IC2 slightly promoted the generation of ROS compared to the control, but there was no significant difference; however, 20 μM IC2 significantly promoted the generation of ROS, which was consistent with the decreasing trend of MMP (Fig. 6D, E). Therefore, we concluded that high-concentration IC2 reduces cellular MMP, damages mitochondrial function, causes excessive ROS production and induces cell apoptosis.

### IC2-induced antiproliferation effect and apoptosis depends on the inhibition of SCD1 in MCF-7 cells

**Fig. 7:**
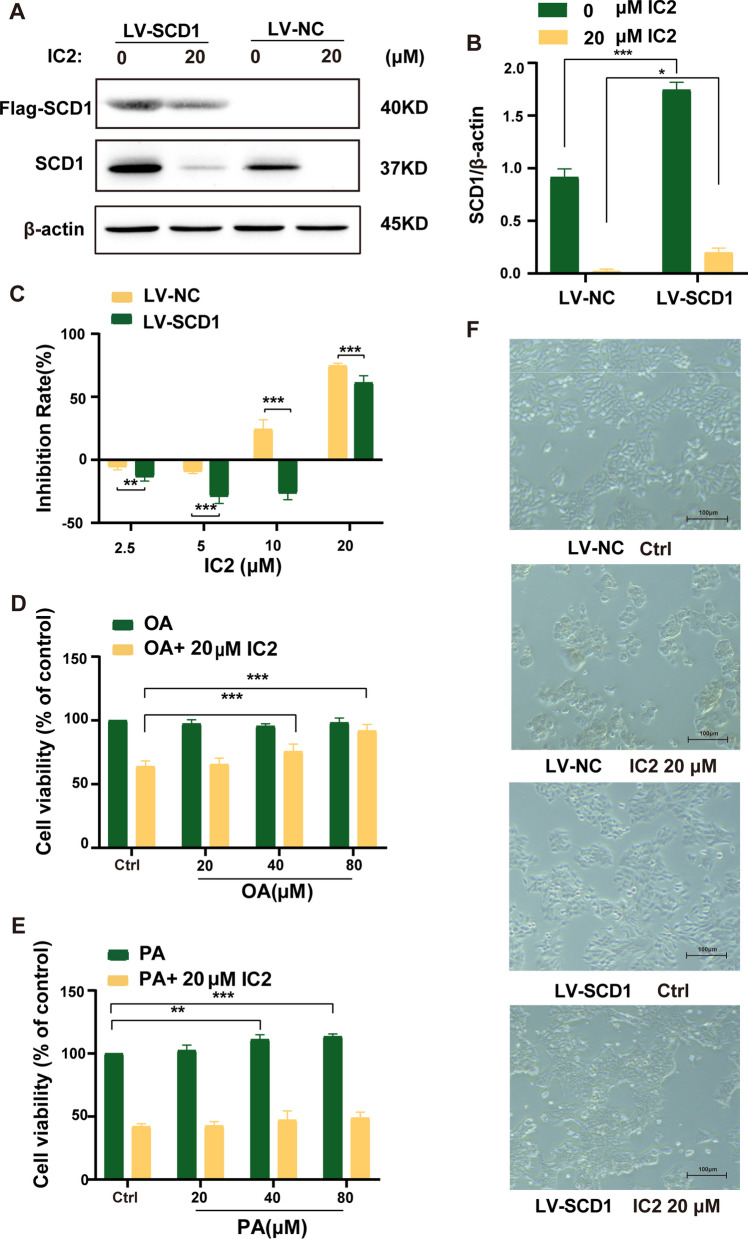
IC2-induced antiproliferation effect depends on the inhibition of SCD1 in MCF-7 cells. **A** IC2 treatment inhibited both total (SCD1 antibody) and exogenous (Flag antibody) SCD1 expression in LV-SCD1 cells. **B** Quantification of the protein expression levels of SCD1 in **A**. **C** SCD1 overexpression alleviated IC2-induced cell growth inhibition. **D** OA rescued the reduced cell viability induced by IC2. **E** PA did not rescue the IC2-induced reduced cell viability. **F** Observation under the microscope at ×50 magnification found that overexpression of SCD1 rescued the IC2-induced reduced cell viability. Data are presented as the mean ± SEM of three independent experiments (n = 3). *P < 0.05, **P < 0.01 and ***P < 0.001 vs. control

Because IC2 significantly reduced the expression of SCD1, we investigated whether this significant reduction leads to IC2-induced antiproliferation effect and apoptosis. SCD1-overexpressing MCF-7 cell line (LV-SCD1) and corresponding normal MCF-7 cell line (LV-NC) were constructed, as shown in Fig. 7A and B, the exogenous (Flag-SCD1) and total expression of SCD1 were detected by flag and SCD1 antibody, respectively. Our data showed that 20 μM IC2 treatment reduced both exogenous and total expression of SCD1. MTT assay showed that treatment of LV-SCD1 cells with IC2 had a significant growth-promoting effect compared to LV-NC cells. (Fig. 7C). In accordance with the MTT assay results, morphological observation also showed that LV-SCD1 cells attenuated cancer cell growth inhibition after treatment with IC2 for 24 h (Fig. 7F). OA is the main product of SCD1, and the decrease in SCD1 expression caused by IC2 prompted us to investigate whether the addition of exogenous OA reverses the cell death induced by IC2. The results showed that the addition of OA increased the viability of IC2-treated cells in a dose-dependent manner, and the cell viability was almost completely reversed when the OA concentration was 160 μM compared to cells without OA (Fig. 7D), while there was no significant change in cell viability compared to the addition of PA, the substrate of SCD1 (Fig. 7E). These results indicated that the death of MCF-7 cells induced by IC2 was dependent on the inhibition of SCD1 expression and activity.

**Fig. 8:**
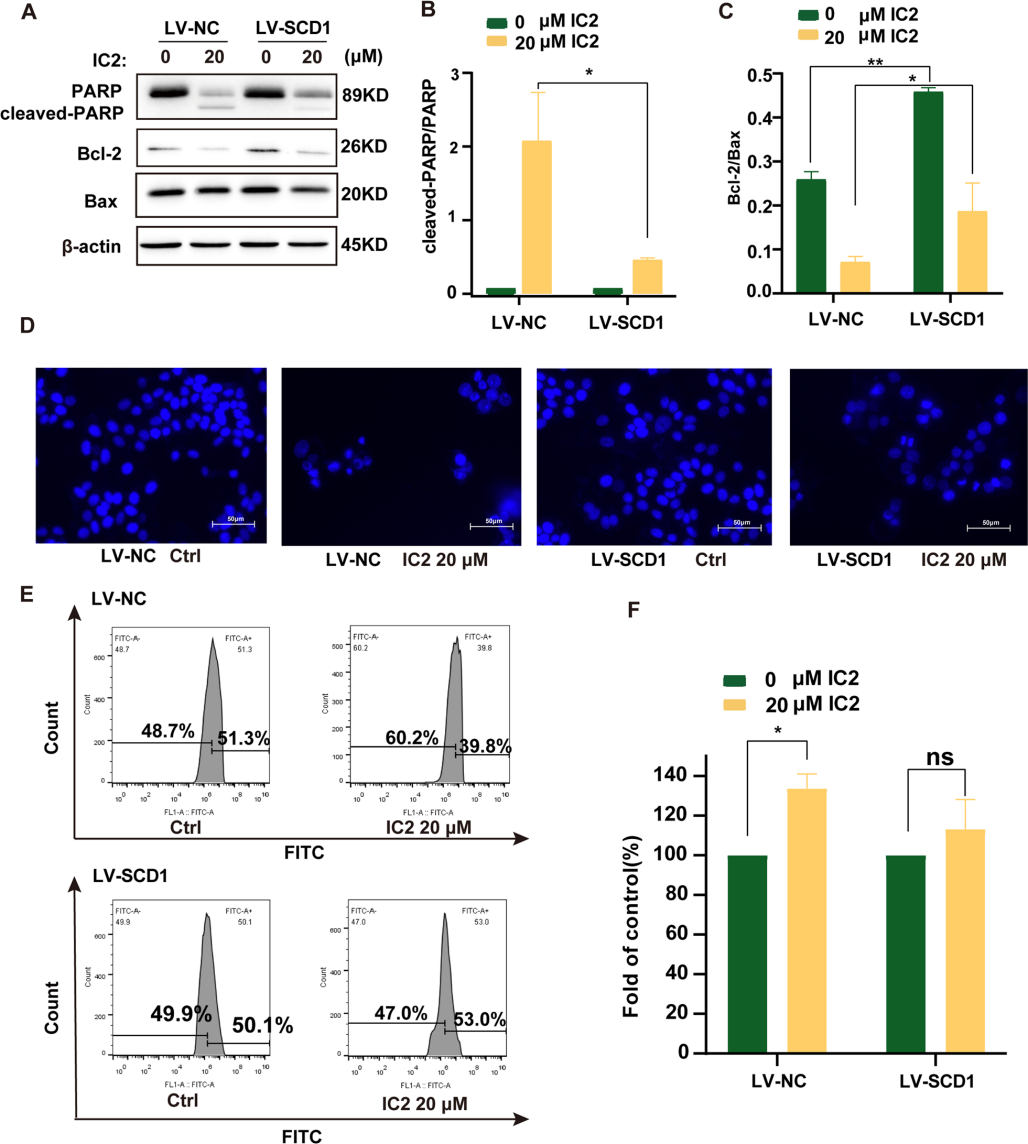
IC2-induced apoptosis depends on the inhibition of SCD1 in MCF-7 cells. **A** Overexpression of SCD1 alleviated the decrease in the protein levels of PARP and Bcl-2. **B**, **C** Quantification of the protein expression levels of cleaved-PARP/PARP and Bcl-2/Bax. **D** Representative images of DAPI staining at ×200 magnification in MCF-7 cells. Overexpression of SCD1 reduced IC2-induced apoptosis. **E** Flow cytometry was used to detect intracellular ROS. Overexpression of SCD1 reduced IC2-induced intracellular ROS increases. **F** Quantification of the level of ROS production compared to the control. Data are presented as the mean ± SEM of three independent experiments (n = 3). *P < 0.05, **P < 0.01 and ***P < 0.001 vs. control

To verify whether this form of SCD1-dependent cell death is apoptosis, SCD1-overexpressing MCF-7 cells were treated with high concentrations of IC2, which significantly alleviated the inhibitory effect of IC2 on PARP and Bcl-2 protein expression (Fig. 8A–C). DAPI staining showed that the number of viable SCD1-overexpressing cells was significantly greater than that in control cells (Fig. 8D). The results also showed that the ROS in the SCD1-overexpressing cells were also significantly reduced compared to control cells (Fig. 8E, F). Thus, the above results indicated that IC2-induced apoptosis in the mitochondrial pathway is dependent on the inhibition of SCD1 in MCF-7 cells.

## Discussion

Breast cancer is currently the tumor with the highest morbidity and mortality in women [1]. Breast cancer is still a vast challenge to public health not only in China but also in other countries in the world. At present, most of the clinical treatments for breast cancer have adverse effects that limit their use, indicating that exploring new therapeutic drugs is still a top priority. The present study developed a new ICT derivative, IC2, which targets SCD1 in breast cancer cells. We found that IC2 induced mitochondrial apoptosis in cells by inhibiting the activity of SCD1, providing molecular evidence that IC2 may be a potential candidate for breast cancer treatment.

Aerobic glycolysis and de novo FA synthesis are two main metabolic aberrations in cancer cells [27, 28]. Considering that lipids are important components of cell membranes and signaling molecules during cancer cell proliferation and progression, it is necessary to target lipogenic enzymes, including SCD1, FASN and ACC, for cancer treatment. Among these targets, SCD1 has recently attracted more attention due to its crucial role in regulating the fatty acid composition of cellular lipids [29]. Indeed, high expression levels of SCD1 have been correlated with cancer cell proliferation, aggressiveness and chemoresistance in various cancer types [30–36]. In addition, silencing SCD1 shows a stronger inhibitory effect than silencing other lipogenic genes but does not affect homologous nonmalignant epithelial cells. Therefore, SCD1 may be a potential target for the treatment of breast cancer [7, 8]. Our study also demonstrated that compared to normal breast tissue, SCD1 expression was relatively higher in most tumor tissues, and the ten-year survival rate of patients with high SCD1 expression in tumor tissues was also significantly lower than that of patients with low SCD1 expression. These results confirmed that SCD1 plays an important role in breast cancer progression.

Recently, a variety of small molecule inhibitors targeting SCD1 have been found to exert antitumor effects, but their side effects have restricted the entry of SCD1 inhibitors into clinical trials. The active ingredients of Chinese herbal medicines have multiple targets and less toxicity, providing new possibilities for cancer therapy. *Epimedii Herba* is a type of angiosperm in the Berberis family, and ICT is the main active ingredient of *Epimedii Herba*. ICT has been demonstrated to exert anticancer effects in liver cancer, breast cancer, prostate cancer and ovarian cancer [16, 17, 37, 38]. Therefore, we sought to develop new SCD1 inhibitors based on modifying the structure of ICT. To fully detect the antitumor activities of ICT derivatives, we studied the inhibitory effects of these compounds on MCF-7 cells and found that IC2 possessed potential antiproliferation effects compared to the other compounds. However, our results also showed that IC2 slightly promoted the growth of MCF-7 cells at low doses, which was similar to ICT due to its estrogen-like effect [11]. Finally, we demonstrated that IC2 had a significant inhibitory effect on the expression and activity of SCD1 in breast cancer cells in a dose-dependent manner.

Our data on the apoptotic effect of IC2 in breast cancer cells supported the well-studied properties of IC2 as an effective anticancer candidate. We found that IC2 induced apoptosis by increasing the level of PARP cleavage products and upregulating the ratio of the antiapoptotic protein, Bcl-2, and the proapoptotic protein, Bax. Previous studies have demonstrated that mitochondria play an important role in apoptosis by regulating the release of pro-apoptotic factors [39], and mitochondrial-dependent endogenous apoptotic pathways are mainly due to the expression of related proteins in the Bcl-2 family, impairing the normal membrane potential of mitochondria, releasing apoptotic factors and triggering the caspase family to have a pro-apoptotic effect [40]. In addition, the accumulation of damaged mitochondria can lead to the massive production of ROS, which are secondary messengers that induce apoptosis and further accelerate mitochondrial destruction. The present study found that IC2 promotes apoptosis by destroying the mitochondrial membrane potential and reducing the expression of the anti-apoptotic factor, Bcl-2, accompanied by the generation of ROS. These results indicated that IC2 induces apoptosis of MCF-7 cells through the mitochondrial pathway.

Studies have shown that the reduction in SCD1 expression promotes autophagy and apoptosis in liver cancer cells [41]. Inhibition of SCD1 promotes PA accumulation and leads to lipid apoptosis, and high expression of SCD1 protects against this apoptosis. Additionally, studies have suggested that exogenous addition of OA eliminates the inhibition of SCD1 silencing on breast cancer cell activity [7]. The present study found that the exogenous addition of the SCD1 product, OA, alleviated the inhibitory effect of IC2 on MCF-7 cell viability, while the addition of the SCD1 substrate, PA, had no significant effect. Interestingly, SCD1 overexpression also weakened the inhibitory effect of IC2 on MCF-7 cells, demonstrating that IC2 inhibits SCD1 activity and reduces the production of protective MUFAs but increases the content of SFAs, which leads to lipotoxicity, resulting in an antitumor effect. We found that overexpression of SCD1 in MCF-7 cells also alleviated the inhibitory effect of IC2 on PARP and Bcl-2 expression as well as reduced the generation of intracellular ROS and reversed the apoptosis of MCF-7 cells. These studies demonstrated that SCD1 participates in IC2-induced cell apoptosis and viability.

In conclusion, we developed a new ICT derivative with anticancer activities and demonstrated for the first time that ICT derivatives promote cell apoptosis by inhibiting SCD1 in breast cancer cells. This study suggested that the ICT derivative, IC2, has certain clinical application potential as a therapeutic drug for breast cancer. Further studies should be conducted to elucidate the detailed mechanism of SCD1 activity and expression regulation in IC2-treated breast cancer cells, and the anticancer effect of IC2 should be investigated in animal models.

## Data Availability

All data generated or analyzed during this study are included in this published article.

## Abbreviations

ICT: Icaritin
SCD1: Stearoyl-CoA desaturase-1
CADD: Computer-aided drug design
SFA: Saturated fatty acid
MUFA: Monounsaturated fatty acids
GC–MS: Gas chromatography-mass spectrometry
DAPI: 2-(4-Amidinophenyl)-6-indolecarbamidine dihydrochloride
IRS: Immunoreactivity score
MMP: Mitochondria membrane potential
ROS: Reactive oxygen species
FAME: Fatty acid methyl esters
OA: Oleic acid
PA: Palmitic acid
